# An Immunohistochemical Survey to Evaluate the Expression of CD105 and CD34 in Ameloblastoma and Odontogenic Keratocyst

**Published:** 2014-12

**Authors:** Shokoofeh Jamshidi, Massoumeh Zargaran, Fahime Baghaei, Setareh Shojaei, Reza Zare Mahmoodabadi, Arash Dehghan, Abbas Moghimbeigi

**Affiliations:** a Dental Research Center, Dept. of Oral and Maxillofacial Pathology, School of Dentistry, Hamadan University of Medical Sciences, Hamadan, Iran.; b Dept. of Oral and Maxillofacial Pathology, School of Dentistry, Hamadan University of Medical Sciences, Hamadan, Iran.; c Dental Research Center, Dept. of Oral and Maxillofacial Pathology, School of Dentistry, Mashhad University of Medical Sciences, Mashhad, Iran.; d Dept. of Pathology, School of Medicine, Hamadan University of Medical Sciences, Hamadan, Iran.; e Modeling of Noncommunicable Disease Research Center, Dept. of Biostatistics and Epidemiology, School of Public Health, Hamadan University of Medical sciences, Hamadan, Iran.

**Keywords:** Odontogenic keratocyst, Ameloblastoma, Angiogenesis, CD105, CD 34

## Abstract

**Statement of the Problem: **Ameloblastoma is the most common odontogenic tumor which is slow-growing, locally invasive and exhibit specific biologic behavior and high recurrence rate. Likewise, odontogenic keratocyst is a developmental odontogenic cyst that has a high recurrence rate and aggressive behavior. There are limited studies considering the relationship between the angiogenesis factors and the biologic behavior of these lesions.

**Purpose:** the aim of this study was to evaluate the mean density of vessels in odontogenic keratocysts and ameloblastoma and investigate its possible relationship with biological behavior of these lesions.

**Materials and Method: **In this descriptive-analytic cross-sectional study, 40 cases, comprising 10 odontogenic keratocysts and 30 ameloblastomas (10 plexiform, 10 follicular, and 10 unicystic type) were selected and were stained immuno-histochemically with CD34 and CD105. The micro vessel density was assessed and compared in all groups. T- test for the independent samples’ One- way Anova, Wilcoxon test and Tukey tests were adopted for statistical analysis.

**Results:** A statistically significant difference was observed in mean vascular density (MVD) between the odontogenic keratocyst and ameloblastoma groups concerning the CD105 and CD34 markers (p= 0.005, p= 0.000, respectively). The MVD was significantly higher in ameloblastomas than odontogenic keratocyst. MVD with CD34 was significantly higher than MVD with CD105 in ameloblastomas (p= 0.00).

**Conclusion: **It can be suggested that angiogenesis might be one of the mechanisms that is more possible to contribute the aggressive biological behaviors in ameloblastoma rather than odontogenic keratocyst.

## Introduction

Ameloblastoma is a tumor arising from odontogenic epithelial cells; it is reported as the most common odontogenic tumor. Ameloblastoma is a slow-growing and locally invasive tumor that has an explicit biologic behavior with high recurrence rate.[1-2] Several microscopic subtypes of ameloblastoma are identified, such as follicular, plexiform, acanthomatous, desmoplastic, granular cell, and basal cell. Among these, the follicular and plexiform are the most prevalent variants.[1-2]

Odontogenic keratocyst (OKC) is a developmental odontogenic cyst that has a high recurrence rate and shows aggressive behavior.[1] Concerning the specific clinicopathological characteristics of OKC, the world health organization (WHO) in 2005, re-classified this lesion as a tumor and renamed it to keratocystic odontogenic tumor.[1, 3] Some research have been performed on studying the invasive behavior and high recurrence rate of ameloblastoma and OKC; whilst only limited studies have adopted stromal factors such as the role of angiogenesis in these lesions.[2, 4-5]

Angiogenesis is defined as the formation of new blood vessels from the existing blood vessels. It occurs in physiologic and pathologic processes including embryogenesis, wound healing, and inﬂammation.[6] Neoplastic tissues require angiogenesis for their growth, development, differentiation, progression, and it also denotes their invasion and metastasis.[7-8] Mean vascular density (MVD) is a quantitative analysis of angiogenesis, which has been evaluated by using various molecules including: CD31, CD34 and CD105 (endoglin).[9-10] The angiogenesis phenomenon has been evaluated in colon, prostate, brain, lung, breast, and cervical tumors.[11]

CD105 is a homodimeric cell membrane glycoprotein and is a component of TGF-β receptor complex. This marker is an indicator of endothelial cell proliferation and is up-regulated during angiogenesis.[12-14] Moreover, the expression of CD105 is one of the most conspicuous characteristics of newly formed blood vessels; its expression is negative or insignificant in previously formed blood vessels, endothelium of the vessels of normal tissues, and the endothelial cells of lymphatic vessels.[15] Some studies showed that CD105 antibody had higher specificity for tumor vessels comparing the other endothelial markers such as: von Willebrand, CD31, CD34 and factor VIII. Therefore, CD105 would be more proper to determine the MVD.[12, 15] Compared to other pan-endothelial markers, CD105 are more commonly implemented in diagnosis, follow-up, determining the treatment response, and the patient’s prognosis.[11, 16-17]

CD34 (Q-BEND 10) is a pan endothelial marker and the cell surface trans-membrane monomeric glycoprotein, which is expressed in the normal and neoplastic endothelial cells of blood vessels. It is employed as a selective vascular marker for the quantitative evaluation of angiogenesis in various lesions regarding its aptitudes and ease of use.[10, 16-22]

MVD is expedient in predicting metastasis and tumor relapse, on the other side, the angiogenesis is crucial for growth, development, differentiation and progression of a tumor. Moreover, ameloblastoma and OKC have same biological behavior and both lesion relapse after treatment.[6-8] Therefore, the aim of this study was to determine the MVD by immuno histochemically adopting CD34 and CD105 in odontogenic keratocysts and ameloblastoma and evaluating any possible relationship between these markers and the biological behaviors of these lesions.

## Materials and Method

In this retrospective cross-sectional descriptive-analytical study, the archived documents of patients referred to the oral pathology department of Hamedan dental school, from 1997 to 2012, were evaluated. All the paraffin blocks, Hematoxylin and Eosin (H&E) stained slides and dental documents of patients with the diagnosis of ameloblastoma odontogenic keratocyst were evaluated by two oral pathologists. Formalin-fixed and paraffin-embedded tissue samples of 10 OKC, 10 plexiform ameloblastoma, 10 follicular ameloblastoma, and 10 unicystic ameloblastoma, with sufficient tissues and proper fixation were included in this study.

The demographic and clinical data including age, gender and the locations of the lesions were recorded. Samples with excessive hemorrhage, severe inflammation, and insufficient tissues were excluded from the study. Slices with 4-µ thickness were prepared from paraffin blocks for immunohistochemical staining. The slides were deparaffinized in xylene and then hydrated in graded alcohol series. For CD105 marker, the endogenous peroxidase activity was blocked by incubating the slides with 3% hydrogen peroxide (H_2_0_2_) in methanol for 30 minutes. For CD34 marker, the slides were incubated in H_2_0_2_ (3 %) in phosphate buffer.

The antigen retrieval for CD34 was performed in a microwave oven at 120^o^C for 10 minutes. The antigen retrieval for CD105 was carried out by treating sections with proteins-K for 5 minutes. To prevent nonspeciﬁc reactions, sections were incubated with 10% serum for 10 minutes. Mouse Anti-Human CD34 Monoclonal antibody (Clone: QBend 10, Product code: M7165 A/S, Glostrup, DAKO, Denmark) and mouse Monoclonal Anti-Human CD105 antibody (Clone: SN6h; Product code: M3527, Dako, North America Inc) were used as primary antibodies in this study. For CD34, the sections were incubated with anti-CD34 antibody at room temperature with a working dilution of 1:50 for 30 min. CD105 antibody was incubated at room temperature for 90 minutes in a humidifying chamber, followed by incubation with secondary biotinylated antibodies and streptavidin for 15 min each. Diaminobenzidine was applied to produce brown staining followed by counterstaining with Mayer’s hematoxylin. After each step; the slides were put in phosphate-buffered solution (PBS). For the negative control, the primary antibody was eliminated and replaced with PBS. The human tonsillar tissue was considered as the positive control.

MVD was assessed using the technique described by Weidner et al.[19] Two experienced oral pathologists evaluated the stained slides under a light microscope (Olympus BX41; Japan) at ×100 and ×400 magnifications. Three areas with the highest amount of vascularization (known as the hot spot) were selected under low magnification (×100) and microvessels were counted in each specimen at ×400 magniﬁcation. The mean number of blood vessels in the three selected regions was considered as the mean vascular density (MVD). The accredited criteria for detecting a microvessel was defined as when a cell or group of cells were colored in brown by CD34 and CD105 markers and were distinctively inside the tumor. The objects which seemed to originate from one blood vessel were counted if they were completely separated from it.[10] Blood vessels with muscular walls were excluded from the study. Independent samples T-test, One-way Anova test and Tukey test were used for statistical analysis of the data by adopting SPSS software version 19. Statistical significance was defined at p≤ 0.05.

## Results

**Figure 1:**
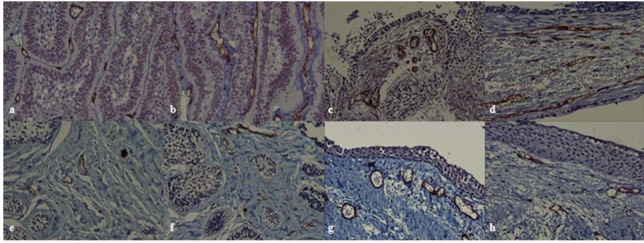
a: Microvessels positive for immunohistochemical staining of CD 105 antigen in ameloblastoma(plexiform type) (×400)  b: Microvessels positive for immunohistochemical staining of CD 34 antigen in ameloblastoma(plexiform type) (×400)  c: Microvessels positive for immunohistochemical staining of CD 105 antigen in ameloblastoma(unicystic type) (×400)  d: Microvessels positive for immunohistochemical staining of CD 34 antigen in ameloblastoma(unicystic type) (×400)  e: Microvessels positive for immunohistochemical staining of CD 105 antigen in ameloblastoma(follicular type) (×400)  f: Microvessels positive for immunohistochemical staining of CD 34 antigen in ameloblastoma(follicular type) (×400)  g: Microvessels positive for immunohistochemical staining of CD 34 antigen in odontogenic keratocyst (×400)  h: Microvessels positive for immunohistochemical staining of CD 105 antigen in odontogenic keratocyst (×400)

10 patients with follicular ameloblastoma, consisting of 6 men (60%) and 4 women (40%), with the age range of 20‒76 years and a mean age of 56.9±16.44 years; 10 patients with plexiform ameloblastoma, consisting of 6 men (60%) and 4 women (40%), with the age range of 18‒67 years and a mean age of 49.7±18.5 years; 10 patients with unicystic ameloblastoma, consisting of 6 women (60%) and 4 men (40%), with an age range of 18‒52 years and a mean age of 37.5±11.5 years; and 10 patients with OKC, consisting of 5 men (50%) and 5 women (50%), with an age range of 13‒76 years and a mean age of 33.8±19.29 years were included in this study. The most common location of the lesions was the posterior mandible. The data regarding the location of the lesions are summarized in Table 1. All the samples (100%), evaluated in 4 groups, were stained positively for CD34 and CD105 markers (Figure 1).

**Table 1:** Location of the studied groups

**Group**	**Frequency**	**Location**			
	**Anterior ** **maxilla**	**Anterior ** **maxilla**	**Anterior ** **mandible**	**Posterior ** **maxilla**	**Posterior ** **mandible**
Ameloblastoma (plexiform)	1(10%)	1(10%)	0(0%)	1(10%)	8(80%)
Ameloblastoma (follicular)	1(10%)	1(10%)	0(0%)	1(10%)	8(80%)
Ameloblastoma (unicystic)	0(0%)	0(0%)	0(0%)	3(30%)	7(70%)
OKC	1(10%)	1(10%)	0(0%)	0(0%)	9(90%)

**MVD based on CD34 marker**

The MVD values in studied groups are summarized in Table 2. One-way ANOVA showed statistically significant differences in MVD among the four study groups (p= 0.000). Tukey test showed significant differences in MVD between follicular ameloblastoma and OKC (p= 0.000), between plexiform ameloblastoma and OKC (p= 0.000), between unicystic ameloblastoma and OKC (p= 0.000) and between plexiform ameloblastoma and unicystic ameloblastoma (p= 0.049).

**Table 2:** CD34 expression and MVD in studied groups

**Group**	**Frequency**	**MIN**	**MAX**	**Means ± standard deviations**
Ameloblastoma (plexiform)	10	14.5	29	20.4.7±4.1
Ameloblastoma (follicular)	10	14.5	26	19.9±3.8
Ameloblastoma (unicystic)	10	13.3	22	16.3±2.9
OKC	10	5.3	12.5	8.6±2.5

MVD in all the three ameloblastoma groups was 18.9±3.9 and higher than that in the OKC group. It was higher in the plexiform ameloblastoma group compared to the unicystic ameloblastoma group.

One-way ANOVA showed significant differences in MVD among the three ameloblastoma groups (p= 0.036). Tukey tests showed statistically significant differences in MVD only between plexiform ameloblastoma and unicystic ameloblastoma (p= 0.045) with higher MVD in the former. Independent samples t-test showed statistically significant differences in MVD between ameloblastoma groups (undividedly) and the OKC group (p= 0.000). 

**MVD based on CD105 marker**

MVD of study groups based on CD105 marker is summarized in Table 3. One-way ANOVA showed significant differences in MVD among the 4 study groups based on CD105 (p= 0.013). Tukey tests revealed significant differences in MVD between follicular ameloblastoma and OKC (p= 0.015) and between plexiform ameloblastoma and OKC (p= 0.047). MVDs were higher in ameloblastoma groups compared to OKC.

**Table 3:** CD105 expression and MVD in studied groups

**Group**	**Frequency**	**MIN**	**MAX**	**Means ± standard deviations**
Ameloblastoma (plexiform)	10	8.3	24	13.7±4.05
Ameloblastoma (follicular)	10	9.4	20.2	14.47±3.8
Ameloblastoma (unicystic)	10	8.9	17.3	11.8±2.2
OKC	10	5	14.3	9.6±2.9

One-way ANOVA did not reveal any significant differences in MVDs marker among the three ameloblastoma groups (p= 0.23). MVD in three ameloblastoma groups was 13.3± 3.5. Independent samples t-test demonstrated significant differences in MVD between the three ameloblastoma groups (undividedly) and the OKC group (p= 0.005).

**Comparison of MVD, considering CD105 and CD34 markers in OKC**

MVDs, in terms of CD105 and CD34 markers, in OKC were 9.6±2.9 and 8.6±2.5, respectively. Wilcoxon test did not show any significant differences in MVDs de termined by two studied markers (p= 0.86).

**Comparison of MVD, considering CD105 and CD34 markers in different forms of ameloblastoma**

Wilcoxon test revealed significant differences in MVD verified by CD34 and CD105 markers in these lesions (p= 0.00), with higher vascular staining in ameloblastoma with CD34 compared to ameloblastoma with CD105 marker. 

## Discussion

OKC with unique clinical and pathological characteristics has particular concerns.[1] Several studies have evaluated the role of epithelium, stroma and their interactions to investigate the biologic behavior of this lesion.[23]^‒^[27] To the best of our knowledge, limited studies have been performed regarding the angiogenesis factors in OKC and in comparison with ameloblastoma. The results of the present study, considering the positive staining for CD34 and CD105 markers, were consistent with those of studies performed by Gadbail and Hande et al., although they used only CD105 marker for the evaluation of angiogenesis.[17, 28] CD34 is a proteoglycan on the surface of endothelial and bone marrow cells and is considered as an adhesion molecule and as a pan-endothelial marker.[18-20]

Some researchers believe that CD34 cannot make a distinction between the host’s primary blood vessels and the neo-angiogenesis; nonetheless, this marker is widely employed in appraising the vascular density in tumoral lesions.[20, 29]

Some researchers have considered the expression of CD105 as one of the prominent properties of newly formed blood vessels. They believe that this marker has high specificity in assessing tumor angiogenesis compared to pan-endothelial markers.[28-30]

In the present study, MVD, in terms of CD105 marker in ameloblastomas, was less than that in terms of CD34 marker. However, MVDs in terms of CD105 and CD34 markers in OKC were not significantly different. In a study by Yao et al., a significantly higher expression of CD34 was shown in hepatocellular carcinoma metastasis compared to the expression of CD105 marker. They concluded that CD105 antibody is an ideal marker to quantify new and immature blood vessels compared to CD34 marker.[31] This might verify that CD105 stains only the newly formed blood vessels whilst CD34 stains the previously formed blood vessels either.

In the present study, the MVDs with the two CD105 and CD34 markers were significantly higher in ameloblastoma compared to OKC. Since neoplastic tissues require oxygen and nutrients to continue their growth and development, they induce neovascularization. On the other hand, angiogenesis is not only necessary for the growth of the tumor, it is also necessary for cellular metastasis.[7-8] MVD can predict the growth of the tumor, metastasis and patient’s survival and this value is related to the aggressiveness of the tumor.[28] Higher angiogenesis in ameloblastoma compared to OKC might reflect higher tissue metabolism, more aggressive biologic behavior, and greater recurrence and growth rate. The growth of the tumor does not only necessitate an increase in the number of blood vessels; it also depends on factors such as protein molecules expressed in the endothelial cells.

Gadbail et al. evaluated and compared angiogenesis in ameloblastoma, keratocystic odontogenic tumor, dentigerous cyst, and normal mucosa using CD105 marker and reported no significant differences in MVD between ameloblastoma and OKC;[17] their finding is almost different from the results of the present study. This difference might be attributed to the differences in methodology and in the number of samples.

Seifi et al. reported a significantly higher MVD with the use of CD34 in multicystic ameloblastoma compared to keratocystic odontogenic tumor and dentigerous cyst. They reported that MVD was significantly higher in keratocystic odontogenic tumor compared to dentigerous cyst;[20] their finding was almost consistent with the results of the present study.

Alaeddini et al. used immunohistochemistry and CD34 marker and reported a significant increase in MVD in ameloblastoma compared to OKC and dentigerous cyst,[32] which is in line with the results of the present study. Although WHO has classified OKC as an odontogenic tumor, based on the results of the present study and those discussed above, further studies might be necessary to identify the biologic behavior and confirm the tumor nature of OKC. In the present study, MVD, in terms of CD105, was not statistically different among the three ameloblastoma groups, which might reflect that despite the clinical, radiological and histological differences of the lesions, their aggressive biologic behaviors are similar.

Other studies have not shown significant differences in MVD, considering CD105 marker, among follicular, plexiform, and unicystic ameloblastoma; coherent with the results of the present study.[28, 33]

In the current study, there were significant differences in MVD(considering CD34 marker) only between the plexiform and unicystic ameloblastomas; however, CD105 marker did not reveal any significant differences between them. To give explanation for this difference, CD105 preferably reacts to newly formed blood vessels and budding endothelial cells; however, CD34 not only reacts to these blood vessels but also reacts to the tissues of the blood vessels which are trapped within the tumor.[33]

## Conclusion

Based on the results of the present study, it is suggested that angiogenesis might be one of the potential mechanisms involved in the more aggressive biologic behavior of ameloblastoma compared to OKC. Moreover, it is suggested that unicystic ameloblastoma has a biologic behavior similar to that of the solid form. It might be possible in future to control the recurrence and invasion of these lesions by inhibiting angiogenesis. 
